# Effects of early intervention in neuromyelitis optica spectrum disorder patients with seropositive AQP4 antibodies

**DOI:** 10.3389/fimmu.2024.1458556

**Published:** 2024-11-01

**Authors:** Binbin Xue, Jia Li, Dewei Xie, Yiyun Weng, Xu Zhang, Xiang Li, Junhui Xia, Jie Lin

**Affiliations:** ^1^ Department of Anesthesiology, The First Affiliated Hospital of Wenzhou Medical University, Wenzhou, Zhejiang, China; ^2^ Department of Neurology, The First Affiliated Hospital of Wenzhou Medical University, Wenzhou, Zhejiang, China

**Keywords:** neuromyelitis optica spectrum disorder, AQP4-antibody, early intervention, immunosuppressive treatments, annualized relapse rate, EDSS

## Abstract

**Background:**

The impact of early intervention with immunosuppressive treatment (IST) in anti-Aquaporin4-antibody (AQP4-ab) seropositive neuromyelitis optica spectrum disorder (NMOSD) has not been thoroughly evaluated.

**Objective:**

This study aims to assess the effects of early IST intervention in patients with NMOSD.

**Methods:**

This retrospective cohort study included 174 treatments from 137 NMOSD patients seropositive for AQP4-antibody, treated with ISTs such as rituximab, mycophenolate mofetil, azathioprine, or tacrolimus. Multiple statistical analyses, including regression discontinuity design (RDD), kaplan-meier analyze, Cox proportional hazards regression model, were employed to evaluate the effects of early IST intervention on annualized relapse rate (ARR) change, Expanded Disability Status Scale (EDSS) change, and time to next relapse.

**Results:**

A total of 174 treatments from 137 patients were analyzed. Patients exhibited significant improvement in ARR[1.95 vs.0, IQR (0.70-6.0 vs. 0-0.42), p<0.001] and EDSS [3.0 vs. 2.5, IQR (2.0-4.0 vs. 1.0-3.0) p<0.001]after IST, although the ARR change was not significant in patients treated with TAC. Early IST initiation was associated with greater improvements in both ARR and EDSS compared to later initiation. RDD analysis demonstrated a time-dependent effect of ARR-change, indicating greater efficacy with early IST intervention.

**Conclusions:**

Early intervention with ISTs in AQP4-antibody-positive NMOSD patients is associated with better outcomes in terms of reducing relapse rate and improving disability. These findings underscore the importance of early treatment in NMOSD.

## Introduction

Neuromyelitis optica spectrum disorder (NMOSD) is a severe autoimmune condition characterized by recurrent attacks of optic neuritis and myelitis, leading to significant neurological disability (1). NMOSD accounts for about one-third of central nervous system (CNS) inflammatory demyelinating diseases in Asian and non-White populations, where it is more prevalent compared to the United States and Europe (2). The median onset age is 40 years, with a notable female preponderance (1, 3). The identification of anti-Aquaporin4-antibody (AQP4-ab) marked a significant milestone in understanding NMOSD. These pathogenic antibodies target astrocytes, initiating a cascade of aberrant immune responses in the CNS, which is the primary pathogenesis in NMOSD (4, 5). Additionally, pro-inflammatory factors, such as interleukin-6, complement activation, and other immune cells exacerbate the disease process (6).

Transverse myelitis (TM), optic neuritis (ON), area postrema syndrome, cerebral syndrome, diencephalic syndrome, and brainstem syndrome are the identified core syndromes in NMOSD (7). Serial episodes with single or multiple lesions result in neurological dysfunction. The sentinel attack involves ON or myelitis in more than 85% of adult patients (7, 8). About one-fifth and one-third of patients develop permanent visual and motor disabilities, respectively, and approximately 23% of patients require wheelchair dependence, with 9% having died within five years of disease onset (9). The principal goal of NMSOD management is the prevention of relapse due to the relapsing nature and poor prognosis. The urgency of early diagnosis and intervention is vital for patients with NMOSD.

Immunosuppressive agents, including azathioprine (AZA), mycophenolate mofetil (MMF), rituximab (RTX), tacrolimus (TAC), eculizumab, satralizumab and inebilizumab, have been recommended for NMOSD patients and have shown efficacy (10–17). Patients with multiple sclerosis (MS) who receive early intervention of disease-modifying therapies (DMTs) achieve better outcomes (18). A previous study indicated that early RTX treatment was associated with a decline in neurological disability (EDSS) at the last follow-up in NMOSD patients with seropositive AQP4-ab (19). However, the effects of IST used in NMOSD has not been fully estimated. Early intervention with IST is hypothesized to mitigate disease progression and improve long-term outcomes.

The aim of our study is to evaluate the effect of early ISTs in patients with NMOSD.

## Methods

### Participants

This retrospective cohort study collected demographic and clinical data from patients at the First Affiliated Hospital of Wenzhou Medical University. The inclusion criteria were as follows: (1) fulfillment of the 2015 International Panel for NMOSD diagnosis criteria, (2) seropositive for AQP4-ab, (3) treatment with one of the ISTs (RTX, MMF, AZA, TAC), and (4) follow-up for at least 6 months. The exclusion criteria were (1) incomplete medical records, (2) lost to follow-up, (3) seronegative or unknown status for AQP4-ab.

Due to concerns about potential adverse effects, the majority of pregnant women at our center chose to discontinue medication and opt for observation instead, or receive intermittent intravenous immunoglobulin (IVIg) who were excluded from our study. Consequently, this group of patients was not included in the analysis.

### Standard protocol approvals and patient consents

This study was approved by the ethical committee of the First Affiliated Hospital of Wenzhou Medical University. Due to the nature of retrospective study, patient consents were waiver with full anonymity of the included patients.

### Procedure

The data collected included sex, age of onset, demyelinating phenotype at onset and relapses, total numbers of attacks, ARR before and after IST, EDSS at IST initiation and last follow-up, time to next relapse after IST initiation, IST information, and concurrent autoimmune disease (Co-AID). The demyelinating phenotypes were defined as ON, TM, area postrema syndrome, cerebral syndrome, diencephalic syndrome, and brainstem syndrome.

Relapse was defined as new or worsening neurological symptoms for more than 24 hours, accompanied by new or worsening MRI lesions was detected. ARR was calculated as the number of relapses per year. If the onset time to IST initiation was less than six months, the pre-ARR was defined over a 6-month period. EDSS was evaluated by medical data by two different certified neurologists. Medical record of EDSS was not applicable for one patients.

Patients may have received more than one IST due to poor response, and outcomes before and after IST switching were considered as independent events. Patients were prescribed MMF, AZA and TAC at least 6 month, and at least one infusion of RTX.

To identify the possible time effect on outcomes, we divided enrolled patients into several groups according to the time from disease onset to IST initiation: 12 months, 24 months, 36 months, 48 months, 60 months.

The endpoints in our study were ARR-change, time to next relapse after ISTs, and EDSS-change.

### Statistical analysis

Statistical analysis was performed using the Statistical Package for the Social Sciences (version 23.0, IBM, Armonk, NY, USA) and R software (version 4.0.3; R Foundation for Statistical Computing, Vienna, Austria; http://www.r-project.org/). Categorical data are presented as percentages and frequencies. Continuous data are presented as the mean and standard deviation (SD), and ranked data by the median and interquartile range (IQR). The Mann–Whitney U test or Student’s t-test for quantitative data, χ² test or Fisher’s exact test for qualitative data. One-way analysis of variance (ANOVA) was introduced to analysis the difference from three comparisons. Missing data was replaced by the median.

Univariate logistic regression was used to evaluate onset age, sex, Co-AID, EDSS before treatment, onset phenotype, and time from onset to IST initiation. Time to next relapse was analyzed using Kaplan-Meier (KM) analysis. The Cox hazard model was employed to evaluate the risk factors related to the first relapse.

Regression discontinuity (RDD) analyze was introduced to explore the potential time-dependent effects related to favorable prognosis. The RD effect reflects the time effect on the outcome. The cutoff values were set as 12, 24, 36,48, and 60 months, respectively. The primary goal of our analysis was exploratory; we aimed to understand the overall trend and potential effects of early IST intervention without imposing strict constraints that might limit the scope of our findings, bandwidth was not set in our analyses. Robustness analysis was performed following RDD.

Restricted cubic spline (RCS) analyze was applied to evaluate the time-response relationship after ISTs and explore the potential linear or non-linear relationship of time from onset to ISTs and outcomes. Multivariable adjusted analyses with 5 knots (12, 24, 36, 48, 60 months) were used. The test result for nonlinearity was first checked. If the test for nonlinearity was not significant, the test result for overall association and linearity was checked, with a significant result indicating the linear association.

Statistical significance was set at two-tailed p < 0.05. Odds ratio (OR), hazard ratio (HR), and the associated 95% confidence interval (CI) values were calculated.

### Sensitivity analysis

Sensitivity analysis were implemented as follows: (1) subsamples of female patients to exclude the sex bias, (2) subsamples of patients using MMF to exclude the possible efficacy bias, (3) subsamples of patients whose ARR before ISTs is less than 6 to exclude the possible relapse rate bias.

## Results

**Figure 1:**
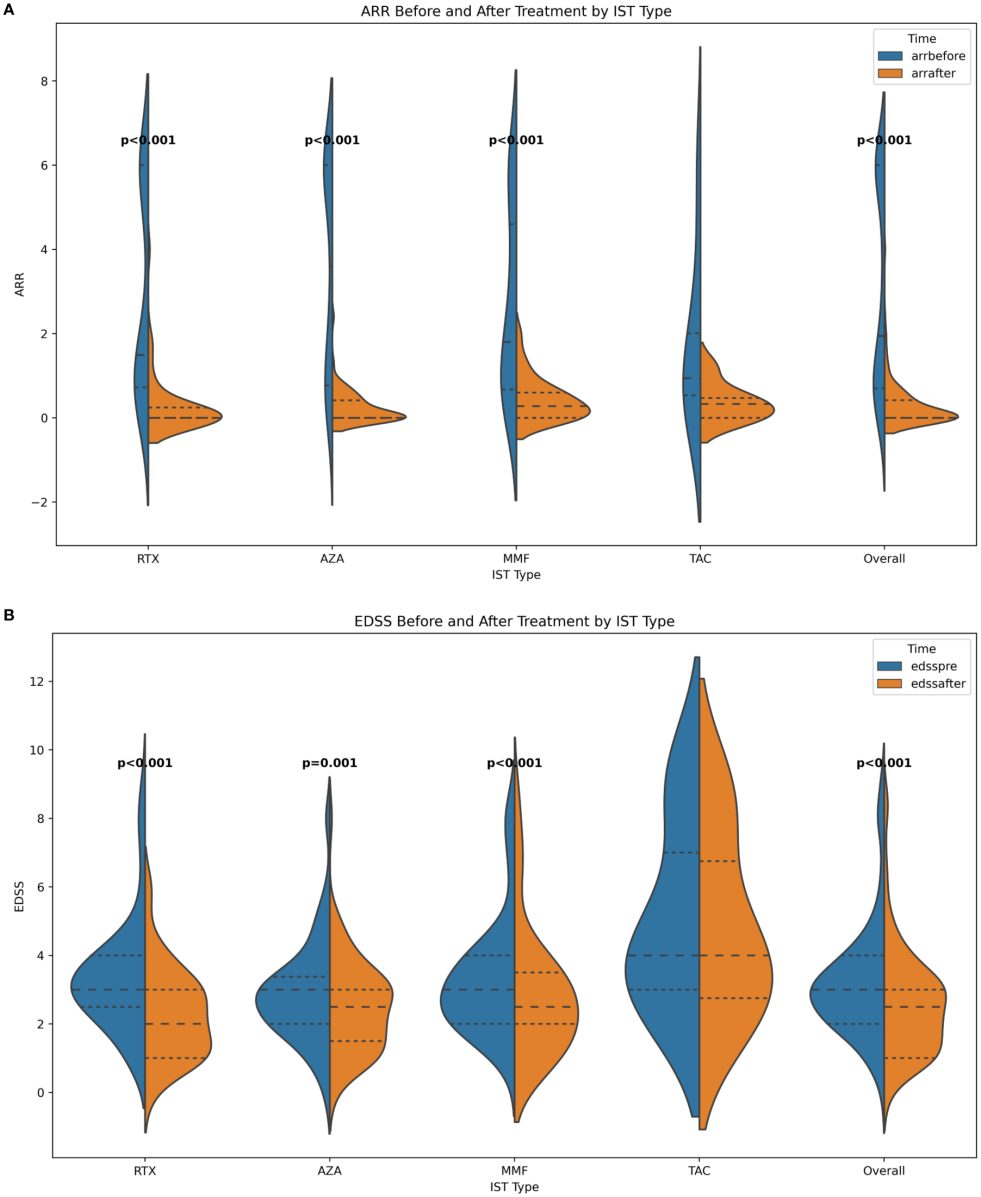
**(A)** ARR before and after ISTs; **(B)** EDSS before and after ISTs. ARR, annualized relapse rate; EDSS, Expanded Disability Status Scale; AZA, azathioprine; RTX, rituximab; MMF, mycophenolate mofetil; TAC, tacrolimus.

A total of 174 treatments from 137 patients were enrolled in our study. Treatments from female patients were 156. The median age at onset was 40 years (range 15-74 years). There were fifty-one patients with NMOSD who also have Co-AID. The median time from onset to ISTs initiation was 14 months (range 0-214 months). Seventy-four relapses were reported (42.53%) after IST. The median following time of RTX, MMF, and AZA were 31.5 (3-106), 32 (6-94), and 32.5 (6-158) months, respectively, while the mean time of TAC was 31.7 (10-73) months. A decreased ARR was observed after most treatments (n=159, 90.34%), with one patient remaining stable. Improvement of neurological disability was observed in ninety-nine episodes (56.25%) after treatments. Both ARR[1.95 vs.0, IQR (0.70-6.0 vs. 0-0.42), p<0.001] and EDSS [3.0 vs. 2.5, IQR (2.0-4.0 vs. 1.0-3.0) p<0.001] at last follow-up were significantly decreased after ISTs (**Figure 1**, **Table 1**).

**Table 1:** Demographic characteristic.

Characteristic	Treatments episodes (n=174)	
Patients, n	137	
Sex		
Female ratio (%)	156/174 (89.7%)	
Male ratio (%)	18/174 (10.3%)	
Co-AID, n	51	
Onset age, median (range), years	40 (15-74)	
TOI, median (range), months	14 (0-214)	
Relapse after ISTs, n(%)	74 (42.53%)	
	Before ISTs	After ISTs
ARR, median (IQR)	1.95 (0.70-6.0)	0 (0-0.42)
RTX	1.50 (0.72-6.0)	0 (0-0.24)
MMF	3.6 (0.77-6.0)	0 (0-0.42)
AZA	1.8 (0.67-4.6)	0.28 (0-0.6)
TAC	0.94 (0.54-2.01)	0.33 (0-0.47)
EDSS, median (IQR)	3.0 (2.0-4.0)	2.5 (1.0-3.0)
RTX	3.0 (2.5-4.0)	2.0 (1.0-3.0)
MMF	3.0 (2.0-3.38)	2.5 (1.5-3.0)
AZA	3.0 (2.0-4.0)	2.5 (2.0-3.5)
TAC	4.0 (3.0-7.0)	4.0 (2.75-6.75)

ARR, annualized relapse rate; AZA, azathioprine; Co-AID, cocurrent autoimmune disease; EDSS, Expanded Disability Status Scale; IST, immunosuppressive treatment; IQR, interquartile range; MMF, mycophenolate mofetil; RTX, Rituximab; TAC, tacrolimus; TOI, Time from onset to IST initiation.

### Effect of early ISTs on ARR-change

The change of ARR after ISTs is a vital endpoint for evaluating the effectiveness in the study of NMOSD. In our study, the ARR of the whole cohort was significantly reduced after ISTs [1.95 vs 0, IQR (0.70-6.0 vs. 0-0.42), p<0.001], as well as in subgroups [RTX: 1.50 vs. 0, IQR (0.72-6.0 vs. 0-0.24), p<0.001; MMF: 3.6 vs. 0, IQR (0.77-6.0 vs. 0-0.42), p<0.001; AZA: 1.8 vs. 0.28, IQR (0.67-4.6 vs. 0-0.6), p<0.001]. However, patients treated with TAC did not show this declining trend [0.94 vs. 0.33, IQR (0.54-2.01 vs. 0-0.47)] (**Figure 1**).

**Figure 2:**
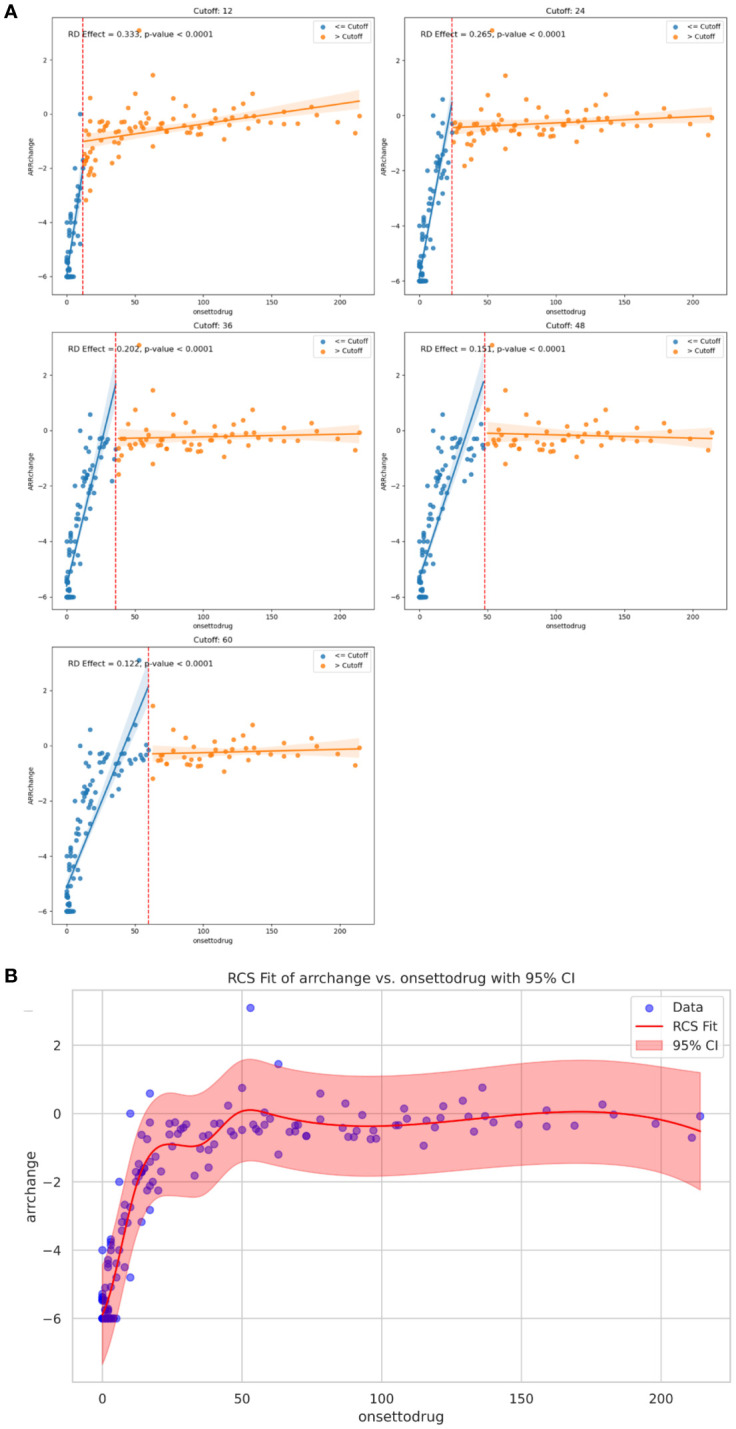
**(A)** Regression Discontinuity plot: RD effects and p value at specific cutoff value of 12, 24, 36, 48, 60 months on ARR change, respectively. **(B)** Curve from the restricted cubic spline regression depicting time-dependent efficacy of ARR change with the IST initiation. ARR, annualized relapse rate; IST, immunosuppressive treatment.

Univariable linear regression analysis revealed no linear correlation between ARR-change after ISTs and time from onset to IST initiation (p>0.05). We then introduced RDD analysis, a quasi-experiment evaluation, to access the potential correlation between the early intervention of ISTs and ARR-change. We assumed the time point of ISTs initiation at 12, 24, 36, 48, 60 months from onset, and the RD effects were -4.58 (95%CI: -0.37- -4.26, p<0.001), -4.06 (95%CI: 0.074- -3.59, p<0.001), -3.84 (95%CI: 0.21 - -3.29, p<0.001), -3.65 (95%CI: 0.36- -3.03, p<0.001), -3.31 (95%CI: 0.39- -2.58, p<0.001), respectively. The RD effects gradually increased with the time from onset to ISTs initiation, indicating the reduced efficacy of ARR-change with the longer initiation time (**Figure 2**). Robustness analysis revealed the approximate tendency (p<0.001). A non-linear correlation between ARR-change and time from onset to IST was described by RCS visualization, with a p-value <0.001 overall (**Figure 2**). The non-linear relationship revealed the time-related efficacy in the early age of disease.

### Time to next relapse

Time to next relapse after ISTs was a commonly used endpoint in the study of NMOSD. Seventy-four relapses were reported during the follow-up. To evaluate the effect of the time from onset to ISTs on the time to next relapse, we introduced Kaplan-meier survival analyze to access the risk factors, including sex, age of onset, Co-AID, lesion on onset, IST types, and time from onset to IST initiation (12, 24, 36, 48, 60 months). Patients treated with RTX had a longer duration to next relapse compared to other ISTs (log-rank test: vs. MMF: p=0.018; vs. AZA: p<0.001; vs. TAC: p=0.043) (**Figure 2**). Patients who received ISTs before 12 (Log-rank test: p=0.053; Breslow test: p=0.047; Tarone-Ware test: p=0.049), 24 (Log-rank test: p=0.020; Breslow test: p=0.045; Tarone-Ware test: p=0.033), 36 (Log-rank test: p=0.009; Breslow test: p=0.047; Tarone-Ware test: p=0.025), 48 (Log-rank test: p=0.009; Breslow test: p=0.052; Tarone-Ware test: p=0.025) months from onset had a longer duration compared to those who received ISTs after these time points, respectively. However, patients in the 60-months group did not show statistical significance.

Poisson regression and Logistic regression indicated that the use of ISTs was highly related to longer remission after treatment (p<0.05). However, time from onset to IST initiation did not influence the remission after ISTs (p>0.05).

RDD analyze was performed using data from relapsed patients after ISTs. Even though the results indicated a declining trend of RD effect with the increasing of cutoff value, there were no statistical differences.

### Effect of early ISTs on EDSS

The EDSS score, which assesses neurological disability in NMOSD patients, also can reflects treatment efficacy. In our study, patients benefited from ISTs, as evidenced by a significant reduction in EDSS scores [overall: 3.0 vs. 2.5, IQR (2.0-4.0 vs. 1.0-3.0), p<0.001; RTX: 3.0 vs 2.0, IQR (2.5-4.0 vs. 1.0-3.0), p<0.001; MMF: 3.0 vs. 2.5, IQR (2.0-3.38 vs. 1.5-3.0), p<0.001; AZA: 3.0 vs. 2.5, IQR (2.0-4.0 vs. 2.0-3.5), p=0.002; TAR: 4.0 vs. 4.0, IQR (3.0-7.0 vs. 2.75-6.75), p=0.046].

EDSS at the last follow-up was influenced by multiple factors. To further explore the potential relationship with ISTs, univariable linear regression revealed that the time from onset to ISTs (coefficient: 0.003, p=0.033, 95% CI:0-0.007) was related to EDSS change, along with time from onset to ISTs less than 12 months (TOI-12) (coefficient: 0.341, p=0.036, 95% CI:0.023-0.658), and time from onset to ISTs less than 24 months (TOI-24) (coefficient: 0.372, p=0.024, 95%CI: 0.050-0.694), IST type (coefficient: 0.312, p=0.007, 95%CI: 0.120-0.503), and EDSS before IST (coefficient: -0.263, p<0.001, 95% CI: -0.351- -0.175). However, these effects diminished with increasing time from onset to ISTs (TOI-36: p>0.05; TOI-48: p>0.05; TOI-60: p>0.05). Multivariable regression analysis revealed that ISTs (p<0.001), EDSS before ISTs (p<0.001), time from onset to ISTs (p=0.021), TOI-12 (p=0.023), and TOI-24 (p=0.004) were associated with EDSS-change after ISTs (detail in **Table 2**).

**Table 2:** Factors affecting the EDSS-change in patients with NMOSD.

	Univariable			multivariable		
	coefficients	95% CI	P value	coefficients	95% CI	P value
IST	**0.312**	**0.120- 0.503**	**0.007**	**0.347**	**0.176- 0.518**	**<0.001**
Sex	0.073	-0.452- 0.599	>0.05			
Co-AID	0.143	-0.207- 0.496	>0.05			
EDSS before IST	**-0.263**	**-0.351- -0.175**	**<0.001**	**-0.281**	**-0.364- -0.197**	**<0.001**
Attack numbers before IST	0.072	-0.001- 0.145	>0.05			
Time from onset to IST initiation	**0.003**	**0.000- 0.007**	**0.033**	**0.003**	**0.000-0.006**	**0.021**
TOI12	**0.341**	**0.023- 0.658**	**0.036**	**0.322**	**0.045- 0.599**	**0.023**
TOI24	**0.372**	**0.050-0.694**	**0.024**	**0.411**	**0.132-0.690**	**0.004**
TOI36	0.324	-0.005- 0.659	>0.05			
TOI48	0.296	-0.050- 0.644	>0.05			
TOI60	0.177	-0.189- 0.545	>0.05			

ARR, annualized relapse rate; Co-AID, cocurrent autoimmune disease; EDSS, Expanded Disability Status Scale; IST, immunosuppressive treatment; TOI, Time from onset to IST initiation.

P-value < 0.05 on multivariate analysis appears as bold.

However, time-dependent efficacy of EDSS-change was not observed from the RDD analysis.

### Sensitivity analysis

**Figure 3:**
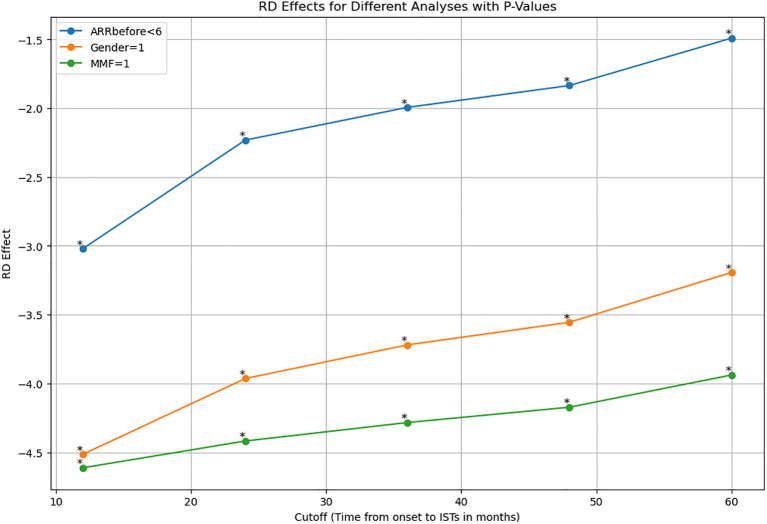
Sensitive analysis indicated RD effects were reduced with time increasing in subsamples of ARR prior to treatment less than 6, female patients, and patients with MMF. ARR, annualized relapse rate; MMF, mofetil mycophenolate. *: p value <0.001.

We applied for sensitivity analysis on ARR-change on subsamples. Results indicated RD effects were reduced with time increasing. Female patients show the same trend along with the whole cohort (p<0.05). MMF-data robustness analysis (p<0.05). ARR before less than 6 (p<0.005) (**Figure 3**, **Table 3**).

**Table 3:** Sensitive analysis for ARR-change.

	Female		MMF		Pre-ARR <6	
Cutoff value (months)	RD effect	P value	RD effect	P value	RD effect	P value
12	-4.51	<0.001	-4.61	<0.001	-3.02	<0.001
24	-3.96	<0.001	-4.42	<0.001	-2.23	<0.001
36	-3.72	<0.001	-4.28	<0.001	-1.99	<0.001
48	-3.55	<0.001	-4.17	<0.001	-1.83	<0.001
60	-3.19	<0.001	-3.94	<0.001	-1.49	<0.001

ARR, annualized relapse rate; MMF, mycophenolate mofetil.

## Discussion

Our study revealed the time-dependent efficacy of early intervention of ISTs. The reduction in ARR tapered off as the time from onset to IST initiation increased. A similar trend was observed in EDSS-change after ISTs, although this did not reach statistical significance. These findings suggest that early IST intervention may reduce the risk of recurrence.

The median onset age of NMOSD is 40-year with a female preponderance, with approximately 80% of patients are seropositive for AQP4-ab. More than 90% of NMOSD patients have a relapsing course, frequently suffering moderate-to-severe neurological deficits (1, 3, 7, 8, 20). About one-fifth of NMOSD patients require wheelchair dependence, and one-third develop visual disability within 5 years of disease onset (9). The poor prognosis of NMOSD necessitates more efficient treatments. High dose of glucocorticoids, plasma exchange, intravenous immunoglobulin, and immunoadsorption are recommended in the treatment of the acute phase (7). The principal goal of disease management is to prevent relapse due to the accumulation of disabilities. B-cell mediated humoral immunity plays a vital role in NMOSD because of the detection of AQP4-ab (4–6, 21, 22).

New therapeutic drugs have gradually emerged with a deeper understanding of the pathogenesis. AZA, MMF, TAC, RTX, eculizumab, inebilizumab, and satralizumab have been proved effective in improvement of ARR and EDSS in NMOSD. Results from our cohort showed similar efficacy of ISTs.

Patients with NMOSD commonly experience a cluster of attacks shortly after disease onset, particularly during the early stage of disease. Due to the nature of NMOSD, early diagnosis and treatment are crucial. However, there are few studies, including randomized controlled trails, focused on the time-dependent efficacy of early intervention. One study reported the long-term disability of AQP4-ab-positive NMOSD patients were reduced by the early RTX treatment.

Our study firstly reported that patients have a greater improvement of ARR with early intervention after disease onset as described in figure. NMOSD is recognized as a rare disease, RCTs based on the time point are not available and may be unethical in real world practice. RDD analysis, a quasi-experiment evaluation, was introduced to reveal time-dependent effect. This statistical approach may lead better understand fore researchers from the real-world experiences.

However, the other commonly used endpoint in NMOSD studies, time to next relapse, did not show the time-dependent effect. Lastly, we evaluated the relationship between EDSS-change and time from onset to ISTs initiation.

Humoral immunity plays a fundamental role in NMOSD, from the maturation of B cells to the secretion of pathogenic antibodies (4–6, 21, 22). Plasma cells (PCs) in the bone marrow (BM) are responsible for producing IgG antibodies, which provide long-term immunity (23). AQP4-abs, which are IgG1 antibodies, are secreted by PCs in the BM or mucosa-associated lymphoid tissue (BALT) and are believed to initiate CNS injury in NMOSD (4, 5, 21, 24). It has been demonstrated that B cells in NMOSD are abnormally skewed towards antibody-secreting cells during the early differentiation phase (22).

Long-lived PCs (CD19^-^CD38^hig^CD138^+^ PCs) (LLPCs) residing within the BM and spleen provide survival factors that maintain continuous antibody production for decades without the need for re-stimulation (23). Unlike short-lived PCs, LLPCs do not respond to immunosuppressive agents and B-cell depleting therapies. It has been reported that human BM is enriched for CD19^-^ PCs in systemic autoimmunity (25, 26). CD19 has a broader expression and a more specific pattern during B-cell differentiate than CD20. However, CD19 expression diminishes as LLPCs differentiate (23, 25, 27, 28). Previous studies have reported that some NMOSD patients are resistant to B cell depletion therapy, even anti-CD19 (10, 29, 30). Data from the N-MOmentum study, which focused on the anti-CD19 antibody- inebilizumab, revealed that about 12% NMOSD patients treated with inebilizumab still experience relapse (10). These results may reflect the role of LLPCs in NMOSD from another angle. Hence, early intervention of IST may interfere the development of LLPCs, leading to a better outcome for NMOSD patients.

Co-AID is not uncommon in patients with NMOSD. These patients tend to exhibit a higher recurrence rate during the early stage of the disease. However, both our previous research and the study by Park SY et al. have not observed a significant difference in long-term prognosis between the NMOSD patients with and without Co-AID (19, 31). Consequently, in the present study, we opted to not to conduct a comparative analysis of the ARR and EDSS between these two groups.

From our results, AQP4-ab-seropositve NMOSD patients experienced a better reduction in recurrence with the early IST intervention, which may lead to alleviate the accumulation of neurological disability.

Our findings are of important significance for guiding the clinical treatment of NMOSD. However, there are limitations to our study. The retrospective nature of this study inherently limits the ability to establish causality. Retrospective studies are subject to various biases which may affect the validity of the findings. Prospective studies are needed to confirm our results and provide stronger evidence for the effects of early IST intervention in NMOSD patients. Although our study enrolled a significant number of patients, the sample size remains relatively small, particularly when considering the subgroup analyses for different ISTs. Future studies with larger sample sizes are necessary to validate our results and provide more robust conclusions. Our study focused on the widely used ISTs, such as RTX, MMF, AZA, and TAC, but did not include newer DMTs such as satralizumab and inebilizumab. These emerging therapies have shown promise in the treatment of NMOSD and should be further analyzed in future studies to provide a more comprehensive evaluation of the time-dependent effects of early intervention. In our RDD analysis, we did not set a specific bandwidth. Although the results were acceptable due to the nature of the statistical analysis model, the absence of a defined bandwidth may introduce some uncertainty in the interpretation of the results. Future analyses should consider setting an optimal bandwidth to improve the precision and reliability of the RDD findings. While our study provides valuable insights into the time-dependent effects of early IST intervention in NMOSD patients, these limitations highlight the need for further research. Large-scale, multi-center studies with diverse patient populations and the inclusion of emerging therapies are essential to validate our findings and inform clinical decision-making in NMOSD management.

## Conclusion

Early intervention with ISTs in AQP4-antibody-positive NMOSD patients is associated with better outcomes in terms of reducing relapse rate and improving disability, highlighting the importance of prompt treatment in NMOSD.

## Data Availability

The data analyzed in this study is subject to the following licenses/restrictions: Data sharing is not applicable to this article. Requests to access these datasets should be directed to Jie Lin, linjiewzra@hotmail.com.

## References

[1] Wingerchuk DM Lucchinetti CF. Neuromyelitis optica spectrum disorder. N Engl J Med. (2022) 387:631–9. doi: 10.1056/NEJMra1904655 36070711

[2] Papp V Magyari M Aktas O Berger T Broadley SA Cabre P. Worldwide incidence and prevalence of neuromyelitis optica: A systematic review. Neurology. (2021) 96:59–77. doi: 10.1212/WNL.0000000000011153 33310876 PMC7905781

[3] Jarius S Ruprecht K Wildemann B Kuempfel T Ringelstein M Geis C. Contrasting disease patterns in seropositive and seronegative neuromyelitis optica: A multicentre study of 175 patients. J Neuroinflammation. (2012) 9:14. doi: 10.1186/1742-2094-9-14 22260418 PMC3283476

[4] Lennon VA Wingerchuk DM Kryzer TJ Pittock SJ Lucchinetti CF Fujihara K. A serum autoantibody marker of neuromyelitis optica: distinction from multiple sclerosis. Lancet. (2004) 364:2106–12. doi: 10.1016/S0140-6736(04)17551-X 15589308

[5] Lennon VA Kryzer TJ Pittock SJ Verkman AS Hinson SR. IgG marker of optic-spinal multiple sclerosis binds to the aquaporin-4 water channel. J Exp Med. (2005) 202:473–7. doi: 10.1084/jem.20050304 PMC2212860 16087714

[6] Chihara N Yamamura T. Immuno-pathogenesis of neuromyelitis optica and emerging therapies. Semin Immunopathol. (2022) 44:599–610. doi: 10.1007/s00281-022-00941-9 35635574

[7] Wingerchuk DM Banwell B Bennett JL Cabre P Carroll W Chitnis T. International consensus diagnostic criteria for neuromyelitis optica spectrum disorders. Neurology. (2015) 85:177–89. doi: 10.1212/WNL.0000000000001729 PMC4515040 26092914

[8] Wingerchuk DM Hogancamp WF O'Brien PC Weinshenker BG. The clinical course of neuromyelitis optica (Devic's syndrome). Neurology. (1999) 53:1107–14. doi: 10.1212/WNL.53.5.1107 10496275

[9] Kitley J Leite MI Nakashima I Waters P McNeillis B Brown R. Prognostic factors and disease course in aquaporin-4 antibody-positive patients with neuromyelitis optica spectrum disorder from the United Kingdom and Japan. Brain. (2012) 135:1834–49. doi: 10.1093/brain/aws109 22577216

[10] Cree B Bennett JL Kim HJ Weinshenker BG Pittock SJ Wingerchuk DM. Inebilizumab for the treatment of neuromyelitis optica spectrum disorder (N-MOmentum): a double-blind, randomised placebo-controlled phase 2/3 trial. Lancet. (2019) 394:1352–63. doi: 10.1016/S0140-6736(19)31817-3 31495497

[11] Han M Nong L Liu Z Chen Y Chen Y Meng H. Safety and efficacy of mycophenolate mofetil in treating neuromyelitis optica spectrum disorders: a protocol for systematic review and meta-analysis. BMJ Open. (2020) 10:e040371. doi: 10.1136/bmjopen-2020-040371 PMC7705552 33257483

[12] Tahara M Oeda T Okada K Kiriyama T Ochi K Maruyama H. Safety and efficacy of rituximab in neuromyelitis optica spectrum disorders (RIN-1 study): a multicentre, randomised, double-blind, placebo-controlled trial. Lancet Neurol. (2020) 19:298–306. doi: 10.1016/S1474-4422(20)30066-1 32199095

[13] Traboulsee A Greenberg BM Bennett JL Szczechowski L Fox E Shkrobot S. Safety and efficacy of satralizumab monotherapy in neuromyelitis optica spectrum disorder: a randomised, double-blind, multicentre, placebo-controlled phase 3 trial. Lancet Neurol. (2020) 19:402–12. doi: 10.1016/S1474-4422(20)30078-8 PMC7935419 32333898

[14] Zhang C Zhang M Qiu W Ma H Zhang X Zhu Z. Safety and efficacy of tocilizumab versus azathioprine in highly relapsing neuromyelitis optica spectrum disorder (TANGO): an open-label, multicentre, randomised, phase 2 trial. Lancet Neurol. (2020) 19:391–401. doi: 10.1016/S1474-4422(20)30070-3 32333897 PMC7935423

[15] Wingerchuk DM Fujihara K Palace J Berthele A Levy M Kim HJ. Long-term safety and efficacy of eculizumab in aquaporin-4 igG-positive NMOSD. Ann Neurol. (2021) 89:1088–98. doi: 10.1002/ana.26049 PMC8248139 33586143

[16] Lin J Xue B Li J Zhu R Pan J Chen Z. Comparison of long-term use of low dose rituximab and mycophenolate mofetil in chinese patients with neuromyelitis optica spectrum disorder. Front Neurol. (2022) 13:891064. doi: 10.3389/fneur.2022.891064 35599732 PMC9120916

[17] Paul F Marignier R Palace J Arrambide G Asgari N Bennett JL. International delphi consensus on the management of AQP4-igG+ NMOSD: recommendations for eculizumab, inebilizumab, and satralizumab. Neurol Neuroimmunol Neuroinflamm. (2023) 10:e200124. doi: 10.1212/NXI.0000000000200124 37258412 PMC10231913

[18] Simonsen CS Flemmen HØ Broch L Brunborg C Berg-Hansen P Moen SM. Early high efficacy treatment in multiple sclerosis is the best predictor of future disease activity over 1 and 2 years in a Norwegian population-based registry. Front Neurol. (2021) 12:693017. doi: 10.3389/fneur.2021.693017 34220694 PMC8248666

[19] Park SY Kwon YN Kim S Kim SH Kim JK Kim JS. Early rituximab treatment reduces long-term disability in aquaporin-4 antibody-positive neuromyelitis optica spectrum. J Neurol Neurosurg Psychiatry. (2023) 94:800–5. doi: 10.1136/jnnp-2022-330714 37268404

[20] Wingerchuk DM Lennon VA Lucchinetti CF Pittock SJ Weinshenker BG. The spectrum of neuromyelitis optica. Lancet Neurol. (2007) 6:805–15. doi: 10.1016/S1474-4422(07)70216-8 17706564

[21] Bennett JL O'Connor KC Bar-Or A Zamvil SS Hemmer B Tedder TF. B lymphocytes in neuromyelitis optica. Neurol Neuroimmunol Neuroinflamm. (2015) 2:e104. doi: 10.1212/NXI.0000000000000104 25977932 PMC4426682

[22] Hoshino Y Noto D Sano S Tomizawa Y Yokoyama K Hattori N. Dysregulated B cell differentiation towards antibody-secreting cells in neuromyelitis optica spectrum disorder. J Neuroinflammation. (2022) 19:6. doi: 10.1186/s12974-021-02375-w 34991631 PMC8740356

[23] Halliley JL Tipton CM Liesveld J Rosenberg AF Darce J Gregoretti IV. Long-lived plasma cells are contained within the CD19(-)CD38(hi)CD138(+) subset in human bone marrow. Immunity. (2015) 43:132–45. doi: 10.1016/j.immuni.2015.06.016 PMC4680845 26187412

[24] Chihara N Aranami T Oki S Matsuoka T Nakamura M Kishida H. Plasmablasts as migratory IgG-producing cells in the pathogenesis of neuromyelitis optica. PloS One. (2013) 8:e83036. doi: 10.1371/journal.pone.0083036 24340077 PMC3858367

[25] Mei HE Wirries I Frölich D Brisslert M Giesecke C Grün JR. A unique population of IgG-expressing plasma cells lacking CD19 is enriched in human bone marrow. Blood. (2015) 125:1739–48. doi: 10.1182/blood-2014-02-555169 25573986

[26] Alexander T Cheng Q Klotsche J Khodadadi L Waka A Biesen R. Proteasome inhibition with bortezomib induces a therapeutically relevant depletion of plasma cells in SLE but does not target their precursors. Eur J Immunol. (2018) 48:1573–9. doi: 10.1002/eji.201847492 29979809

[27] Tedder TF. CD19: a promising B cell target for rheumatoid arthritis. Nat Rev Rheumatol. (2009) 5:572–7. doi: 10.1038/nrrheum.2009.184 19798033

[28] Blüml S McKeever K Ettinger R Smolen J Herbst R. B-cell targeted therapeutics in clinical development. Arthritis Res Ther. (2013) 15 Suppl 1:S4. doi: 10.1186/ar3906 PMC3624127 23566679

[29] Collongues N Brassat D Maillart E Labauge P Ouallet JC Carra-Dalliere C. Efficacy of rituximab in refractory neuromyelitis optica. Mult Scler. (2016) 22:955–9. doi: 10.1177/1352458515602337 26362900

[30] Damato V Evoli A Iorio R. Efficacy and safety of rituximab therapy in neuromyelitis optica spectrum disorders: A systematic review and meta-analysis. JAMA Neurol. (2016) 73:1342–8. doi: 10.1001/jamaneurol.2016.1637 27668357

[31] Lin J Xue B Li J Xie D Weng Y Zhang X. The relationship between neuromyelitis optica spectrum disorder and autoimmune diseases. Front Immunol. (2024) 15:1406409. doi: 10.3389/fimmu.2024.1406409 38994358 PMC11236685

